# Adrenal Vein Sampling: The Role of a Diagnostic Inspiratory Contrast-Enhanced CT Scan in Interventional Planning

**DOI:** 10.3390/diagnostics15131716

**Published:** 2025-07-05

**Authors:** Filip Njavro, Erin Kos, Karin Zibar Tomšić, Maja Prutki, Ana Marija Alduk

**Affiliations:** 1 Clinical Department of Diagnostic and Interventional Radiology, University Hospital Centre Zagreb, 10000 Zagreb, Croatia; 2 Department of Anesthesiology, Reanimatology, Intensive Care Medicine and Pain Treatment, General Hospital Pula, 52100 Pula, Croatia; 3 Department of Endocrinology, University Hospital Centre Zagreb, 10000 Zagreb, Croatia; 4 School of Medicine, University of Zagreb, 10000 Zagreb, Croatia

**Keywords:** adrenal veins, primary aldosteronism, catheterization, computed tomography

## Abstract

**Background/Objectives**: Adrenal vein sampling is the gold standard for differentiating between unilateral and bilateral primary aldosteronism and guiding treatment. This study evaluates the utility of inspiratory CT scans in interventional planning, specifically assessing the right adrenal vein visualization and positional discrepancies during fluoroscopy. **Methods**: A retrospective analysis of 133 patients who underwent adrenal vein sampling was performed. Pre-procedural inspiratory CT scans were reviewed for visualization and location of the right adrenal vein using vertebral body levels as reference. The position of the right adrenal vein was then compared with the fluoroscopic findings during adrenal veins sampling. **Results**: The right adrenal vein was visualized on CT scans in 99.2% of patients. Cohen’s kappa demonstrated almost perfect agreement for both visualization of the right adrenal vein and position measurement. A median difference of three vertebral levels was observed between the level of the right adrenal vein on CT and fluoroscopy, with fluoroscopy showing a more cranial position in 91.7% of cases. **Conclusions**: Inspiratory CT scans visualize the right adrenal vein effectively and aid the planning of adrenal vein sampling. Understanding the positional discrepancies caused by respiratory motion is crucial for successful cannulation of the right adrenal vein, minimizing procedure time and contrast consumption and ultimately enhancing patient outcomes in the management of primary aldosteronism.

## 1. Introduction

Primary aldosteronism (PA) is the most common cause of secondary hypertension [1], with a prevalence of ~11% in hypertensive patients [2,3,4]. Adrenal vein sampling (AVS) is the gold standard for diagnosing the PA subtype. The distinction between the subtypes, unilateral and bilateral PA, significantly influences patient management, as unilateral cases may be treated effectively with surgery, while bilateral cases typically require lifelong medical therapy. In patients with PA, lateralization of aldosterone hypersecretion is the main inclusion criterion for unilateral adrenalectomy, with the macroscopic appearance of the gland being irrelevant [5].

AVS is a technically challenging procedure, mainly due to difficulty in cannulating the small (2–4 mm diameter) and usually short right adrenal vein (RAV), which directly enters the inferior vena cava’s (IVC) posterior–lateral aspect. However, some report RAV sharing a trunk with the hepatic veins in up to 10% of cases [6]. RAV cannulation failure significantly limits the diagnostic utility of AVS. Failed AVS procedures result in inconclusive lateralization studies, preventing accurate subtyping of primary aldosteronism and delaying optimal treatment decisions. Patients with unsuccessful AVS may undergo repeat procedures with additional radiation exposure and contrast administration or receive medical therapy instead of potentially curative unilateral adrenalectomy. Although contrast-enhanced CT scan has downsides (radiation exposure and iodine contrast application) compared to non-enhanced MR imaging, it has better spatial resolution and, therefore, better visualization of RAV. The latest expert consensus suggests that contrast-enhanced CT examination prior to AVS helps identify RAV and delineate its anatomy [7]. MR imaging is a helpful alternative when there is a risk of complications due to the application of contrast media or radiation exposure [8].

It has been observed that the craniocaudal level of the RAV orifice can significantly differ during the respiratory cycle due to diaphragm breathing excursions [9]. The craniocaudal location of the RAV orifice in catheter venography is closer to that in an expiratory CT scan than in an inspiratory CT scan [10]. When patients present to our institution for AVS, CT scans for other clinical indications have often been performed previously. Standardized abdominal CT imaging is conventionally performed with an inspiratory breath-hold protocol, likely due to the ability to hold the breath longer during inspiration than during expiration [11]. To minimize additional radiation exposure and exposure to further iodine contrast administration, we prioritize utilizing the existing CT scans for AVS planning. Therefore, we use inspiratory CT scans for AVS planning. While we are aware of the possibility of discrepancies due to respiratory motion during fluoroscopy, performing dedicated expiratory CT scans would deviate from our established protocol and lead to unnecessary variability. In this paper, we will assess the role of diagnostic inspiratory CECT scan in AVS planning. We will evaluate the reliability and reproducibility of an inspiratory CT in visualizing the RAV and quantify the differences in its position compared to fluoroscopic findings.

## 2. Materials and Methods

### 2.1. Patients

This retrospective study at the University Hospital Centre Zagreb included AVS patients between November 2015 and July 2022. Informed consent was waived by the institutional review board due to the study’s retrospective design. Consecutive patients who met the inclusion criteria were selected: successful RAV catheterization (selectivity index ≥ 5), available pre-procedural CT scan, and AVS fluoroscopy series in the picture archiving and communication system (PACS). Patients not fully meeting the established criteria were excluded from the study. The indication for AVS, age, and gender were extracted from the medical records of the included patients.

### 2.2. CT Scan Protocol

Preinterventional imaging was performed using a 128-slice CT scanner (SOMATOM^®^ Definition AS, Siemens Healthineers, Erlangen, Germany) with the following parameters: collimation 128 × 0.6 mm, rotation time 0.5 s, tube voltage 120 kV. Iodinated contrast (Omnipaque^TM^ 300 mg I/mL, GE HealthCare, Chicago, IL, USA) was administered intravenously at 1.5 mL/kg body weight (2–3 mL/s). Scanning occurred 90 s post-contrast injection during breath-hold inspiration. The reconstructed slice thickness was 1.5 mm with a 1 mm overlap.

### 2.3. AVS Procedure

All procedures were performed by one interventional radiologist (AMA). On the day of the AVS, the images were reviewed by the interventional radiologist aiming to recognize and localize the adrenal veins. AVS was performed after an overnight fast using a Shimadzu BRANSIST safire system (Shimadzu Corp., Kyoto, Japan). Patients were positioned supine for at least one hour before sampling. Continuous ACTH stimulation (50 µg/h) started 30 min before sampling and was continued throughout [12]. A 5 F sheath was inserted into the right common femoral vein under ultrasound guidance, and 5 F Tempo^TM^ catheters (Cordis Corporation, Hialeah, FL, USA) were used for selective adrenal vein catheterization. During RAV cannulation, Visipaque^TM^ contrast (270 mg I/mL, GE HealthCare, Chicago, IL, USA) was gently applied under continuous fluoroscopy to prevent rupture of small intraglandular veins. The procedure was performed with the patient breathing freely. Sequential blood samples were taken from the RAV, then from the external iliac vein (via the sheath), followed by the left adrenal vein, and then again from the external iliac vein. AVS was deemed technically successful if the selectivity ratio (SR) was ≥5:1, which is determined by comparing cortisol levels in each adrenal vein to those in the external iliac vein [13].

### 2.4. Evaluation of CECT and AVS Fluoroscopy Series

**Figure 1:**
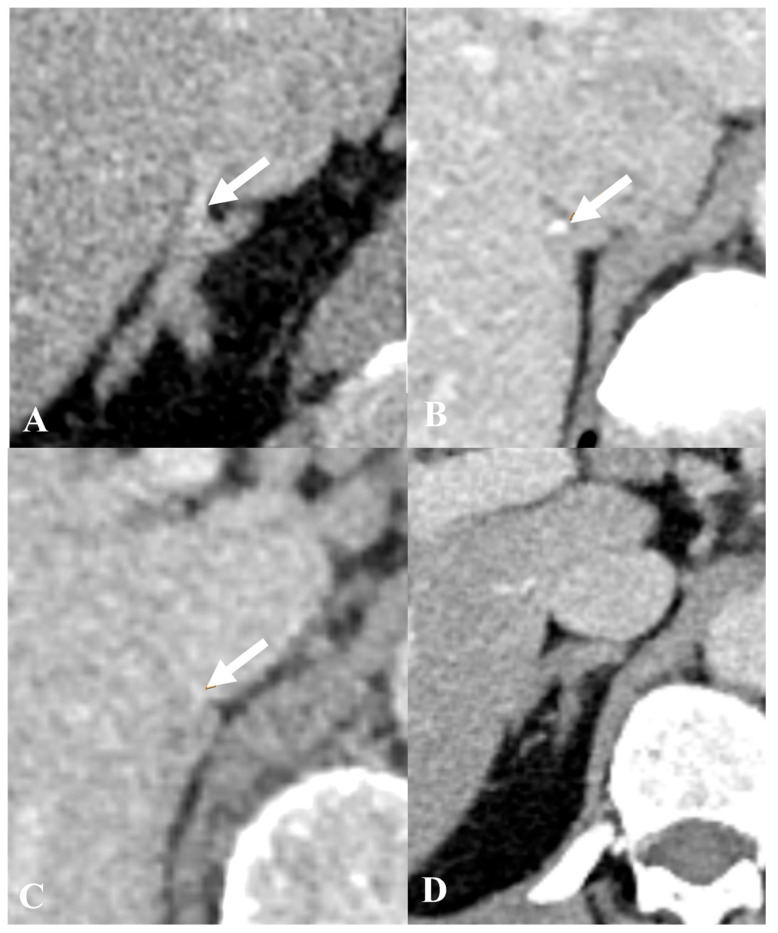
The quality of the RAV visualization on CECT. (**A**) Excellent visualization of the RAV as a tubular structure emanating from the right adrenal gland and entering the IVC. (**B**,**C**) Moderate and poor visualization of the RAV, respectively. (**D**) No visible RAV. The white arrow in (**A**–**C**) indicates the RAV.

The data for this retrospective study were collected at least 6 months after AVS to avoid bias. Two radiologists (AMA, FNJ) with 15 and 5 years of experience independently analyzed images using the hospital’s PACS (Sectra IDS7, Sectra AB, Linköping, Sweden). Visibility of the RAV orifice in the IVC was assessed on the pre-procedural CT and its position on the CT and fluoroscopy. The criteria used to identify RAV on CT scans were taken from Matsuura et al., where RAV is considered to be delineated when a linear or tubular structure is seen emanating from the right adrenal gland and entering the IVC either directly or indirectly [14]. The quality of the RAV visualization on contrast-enhanced CT was scored using a four-point semi-quantitative scale (4, excellent; 3, moderate; 2, poor; 1, not visible), as shown in Figure 1. For the detection of RAV on the AVS fluoroscopy series, the most common morphological patterns described by Daunt were used [15]. This was confirmed retrospectively by SR ≥ 5:1.

**Figure 2:**
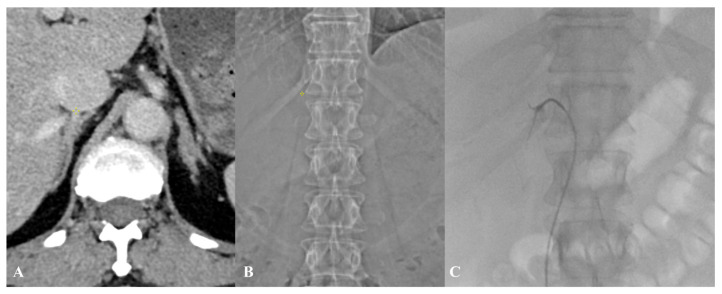
A 45-year-old male with confirmed primary aldosteronism. Using a 3D pointer (yellow symbol) on axial CECT images (**A**) and CT-scout (**B**), the RAV orifice was identified at the T12/L1 intervertebral level (**C**). During fluoroscopic AVS with free breathing, successful cannulation was achieved with a C1 catheter at the second quarter of T12 level, representing a cranial shift of three vertebral levels. The selectivity index achieved was 15.2.

The position of the RAV on CT scans and fluoroscopy series was determined by referencing the vertebral segments, according to the anatomical study by Monkhouse and Khalique [16], with each vertebral body divided into four equal levels in a craniocaudal fashion, with the intervertebral disc as an additional level. In patients with no visible RAV, the center of the adrenal gland was used to approximate the height of the RAV orifice, as suggested by Daunt [15]. The precise location of RAV on the CT image was marked using a three-dimensional pointer, while its height, relative to the vertebral bodies, was determined using the CT-scout performed prior to the CT scan (Figure 2). On AVS fluoroscopy during free breathing, the RAV orifice height was measured at the most cranial location during the normal tidal expiration’s end phase to obtain the greatest difference in height of the same RAV orifice. The difference in RAV orifice height between pre-procedural CT and fluoroscopy was analyzed.

### 2.5. Inter-Rater Reliability Coefficient

After both readers collected the data on RAV orifice heights from the CT scans and AVS fluoroscopy, the inter-rater reliability coefficient (Cohen’s kappa) was calculated for the quality of RAV visualization on CT scans and the measured heights of the RAV orifice on both CT scans and fluoroscopy series. Kappa values were interpreted in accordance with the recommendations set forth by Cohen [17]: ≤0 = no agreement, 0.01–0.20 = none to low, 0.21–0.40 = fair, 0.41–0.60 = moderate, 0.61–0.80 = substantial, and 0.81–1.00 = almost perfect agreement. Significant agreement was defined as kappa ≥ 0.61 (substantial or near perfect).

Statistical analysis was performed using JASP Team software (2024, version 0.19.3). The data were presented as absolute numbers and percentages, means with SD, or medians with interquartile ranges.

## 3. Results

**Figure 3:**
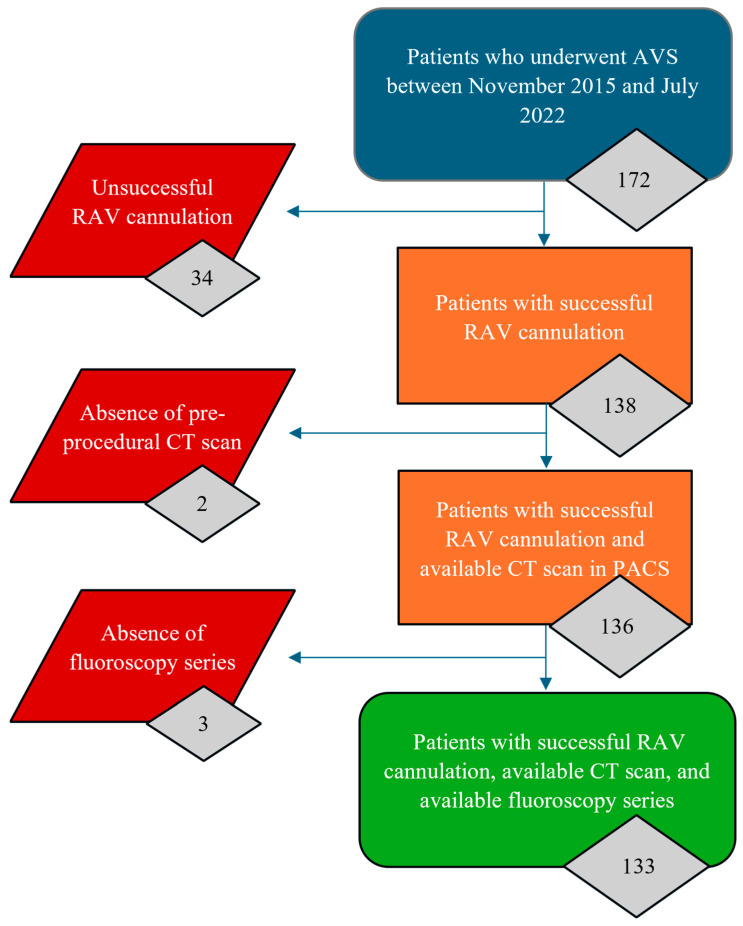
A flowchart depicting the inclusion and exclusion criteria outlining the sequential filtering process used to determine participant eligibility for this study.

A total of 172 AVS procedures were performed between November 2015 and July 2022. As systematically listed in Figure 3, 39 patients were excluded from the study due to the following reasons: unsuccessful RAV cannulation was observed in 34 patients (19.77%), absence of pre-procedural CT scan in PACS was noted in two patients (1.16%), and lack of procedural AVS fluoroscopy series in PACS was identified in three patients (1.74%). Thus, 133 patients were included in the study. The selectivity index for the RAV ranged from 5.1 to 96.6 (median: 29.30). The patient population included 79 men and 54 women, with a median age of 53 years, ranging from 20 to 73 years. Endocrinologically confirmed PA was the indication for AVS in all cases. Four types of catheters were used to successfully cannulate the RAV. Successful cannulation of RAV was achieved in 80/133 patients (60.15%) with a Cobra 1 catheter, in 35/133 patients (26.32%) with a Simmons 1 catheter, in 13/133 patients (9.77%) with a Mikaelsson catheter, and in 5/133 patients (3.76%) with a Simmons 2 catheter. The patient and procedure characteristics are presented in Table 1.

Cohen’s kappa coefficients demonstrated almost perfect inter-reader agreement, with values of 0.836 for RAV orifice visualization on pre-procedural CT scans, 0.878 for RAV orifice levels on pre-procedural CT scans, and 0.818 for RAV orifice levels on procedural fluoroscopy. As there were no significant differences between the readers, the results from Reader 1 are presented in the following text.

The RAV was visualized in 132/133 patients (99.2%). The degree of visualization was considered excellent in 35 patients (26.32%), moderate in 50 patients (37.59%), poor in 47 patients (35.34%), and not visible in one patient (0.75%).

**Figure 4:**
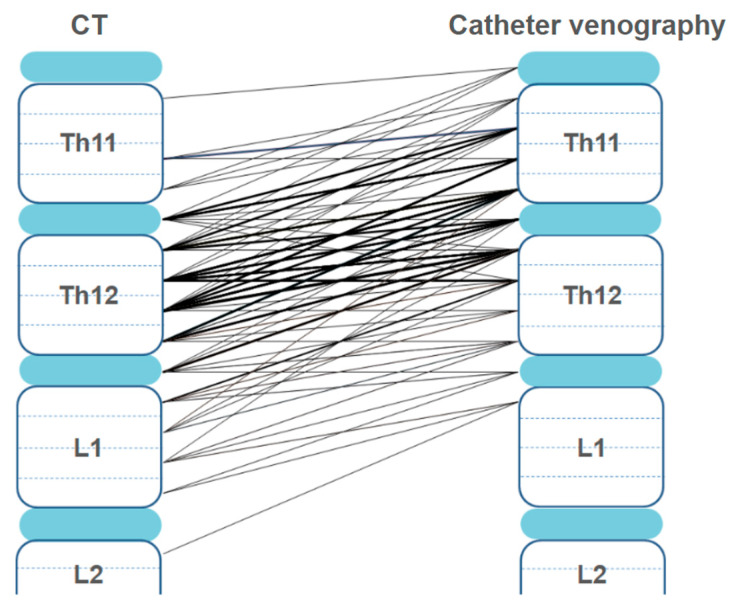
Craniocaudal differences in the height of the RAV orifice measured on inspiratory CECT and catheter venography. The lines between the vertebral bodies connect the RAV orifice heights measured on both modalities in each individual patient, with the line thickness proportional to the number of patients (a thicker line represents more patients in whom the values shown were measured).

**Figure 5:**
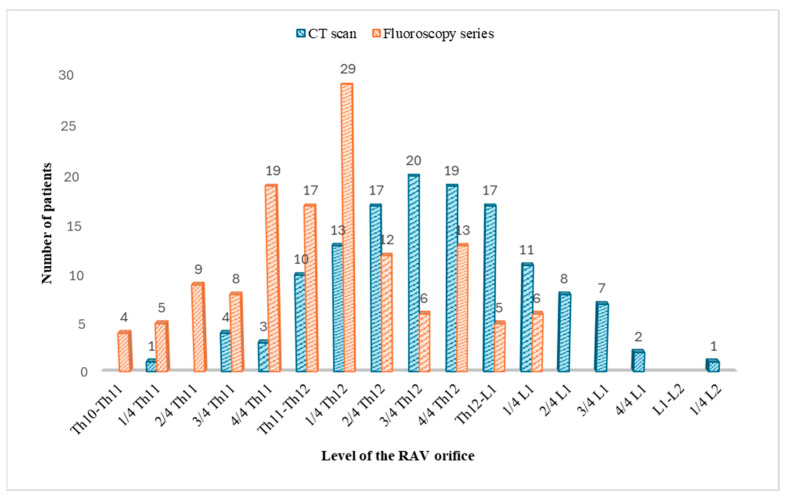
A diagram showing the number of patients with the RAV orifice measured at each vertebral level on both CECT and AVS fluoroscopy.

The results of the measured RAV orifice heights, both on the CECT scans and on the fluoroscopy series, are shown in Figure 4 and Figure 5.

The paired samples *t*-test revealed that the RAV orifice was located significantly higher in the fluoroscopy series than in the inspiratory CT (*p* < 0.001). The median difference in the RAV height between CT and fluoroscopy was three levels (IQR = 2, range 0–8). In 122/133 patients (91.7%), the fluoroscopy height was higher than that on the CT scan. In only two patients (1.5%), lower heights of the RAV orifice were measured on the fluoroscopy series compared to the CT scans: in the first patient, the height of the fluoroscopy-measured RAV orifice was one level beneath the height measured on the CT scan, and in the second patient, it was two levels beneath. In nine patients (6.8%), the heights of the RAV orifice measured on the pre-procedural CT scans were on the exact same level as those measured on the fluoroscopy series.

## 4. Discussion

A contrast-enhanced CT scan should be performed prior to the AVS procedure to more efficiently cannulate the RAV, reduce the radiation time during the procedure, and limit the use of contrast [5,7]. Some centers perform pre-interventional CT scans with breath-hold expiration or with breath-hold without inspiration and expiration, which may reduce the difference in the RAV orifice height between CT scans and AVS fluoroscopy [10,18,19]. However, many patients may have prior CT scans available, which could potentially be utilized for planning, thus minimizing the need for additional radiation exposure. It is crucial to acknowledge that dedicated CT scans for AVS planning in these patients result in unnecessary radiation. Therefore, to minimize radiation and contrast exposure, we prioritize using existing inspiratory CT scans for AVS procedure planning. Although we understand respiratory motion can cause discrepancies during fluoroscopy, deviating from our protocol with dedicated expiratory CT in patients with no previous scans would introduce unnecessary variability. It has been reported in the literature that moving the catheter 1 cm cranially from the RAV orifice height measured on the pre-procedural inspiratory CT scan may assist in efficient RAV cannulation [15], but we found this maneuver to be insufficient in many cases. In addition, some authors suggest that the total maximum vertical movement of the kidneys during respiration and therefore of the adrenal glands could reach approximately 4 cm, which could be a major mismeasurement for RAV cannulation if we plan an intervention according to a CT scan performed in only one phase of the respiratory cycle [20]. In this paper, we aimed to measure the largest differences between the RAV orifice height on pre-procedural inspiratory CT scans and the normal breathing AVS fluoroscopy, which may assist interventionalists in more efficient RAV cannulation, especially in cases of increased respiratory excursions. To our knowledge, there are only two studies that included comparison of inspiratory CT with catheter venography with a small number of patients [10,21].

This study found that the RAV orifice was reliably visualized on CT in most patients (99.2%), supporting the consensus that pre-procedural CT is crucial for RAV anatomy. Our adoption of the criteria of Matsuura et al. [14] aligns with their findings on CT effectiveness. However, the varying degrees of visualization (excellent, moderate, poor, and not visible) underscore the inherent anatomical variability of the RAV, a challenge also noted by Daunt [15].

Furthermore, our analysis of the RAV orifice position on both CT scans and fluoroscopy series revealed a consistent pattern, with the majority of RAV orifices located within the Th11-L1 vertebral levels, which is consistent with the anatomical study by Monkhouse and Khalique [16]. However, we observed a notable discrepancy in the precise vertebral level of the RAV orifice between CT and fluoroscopy, attributable to the caudal movement of the abdominal organs during inspiration due to diaphragmatic contraction and flattening. In most patients, the heights recorded on the pre-procedural CT scans were lower than the heights confirmed on fluoroscopy (Figure 2), confirming the results described by Hara et al. [10]. The median difference in the RAV height between CT and fluoroscopy was three quarters of a vertebral body with the maximum difference of two vertebral bodies, exceeding findings in study by Hara et al. [10]. Interventional radiologists performing AVS should recognize this substantial variation when relying on inspiratory CT for procedural planning and start with a cranial approach: when initiating the fluoroscopic search for the RAV, one should begin approximately three quarters of a vertebral body above the RAV level indicated on the pre-procedural CT scan. This helps to account for the potential cranial shift of the RAV during fluoroscopy. If the RAV remains unidentified within a range of 0 to 3/4 of a vertebral body cranially from the CT-determined position, one should proceed with a more cranial search, extending up to two vertebral levels above the CT-indicated location, to ensure a comprehensive exploration.

In only nine patients (6.8%), the heights of the RAV orifice measured on the pre-procedural CT scans were on the exact same level as those measured on the fluoroscopy series, which could be explained either by weaker inspiration for a breath-hold CT scan or reduced diaphragm excursion during normal respiration.

The high inter-rater reliability coefficients for the RAV visualization and orifice height measurements demonstrate the reproducibility and consistency of our findings, reinforcing the objectivity of our assessment.

The successful cannulation rates achieved with the different catheter types provide valuable insights into the technical aspects of AVS. The C1 catheter demonstrated the highest success rate, suggesting its suitability for RAV cannulation. This is consistent with anecdotal observations in clinical practice, though direct comparisons across studies are limited due to varying techniques and patient populations.

Our study had limitations. First, RAV sampling was unsuccessful in 34 patients, and CT/AVS fluoroscopy data were unavailable for five others. Consequently, the position of the RAV orifice verified by pre-procedural CT scan and catheter angiography could not be correlated in these patients. Thus, only 133 patients were evaluated, potentially biasing the analysis. The relatively small size of our dataset may limit the broader applicability and generalizability of our findings to other populations or clinical settings. Secondly, while all patients enrolled in the study were instructed to take a deep breath during pre-procedural CT scan, breath quality was not assessed regarding maximal inhalation and potential further RAV orifice movement. Finally, it was not possible to assess normal breathing quality during AVS fluoroscopy. Patient discomfort and unfamiliarity with the procedure may alter breathing patterns (deeper/more frequent vs. shallower/less frequent), affecting diaphragmatic excursions and RAV orifice movement.

Despite these limitations, our study strongly supports the use of pre-procedural inspiratory contrast-enhanced CT prior to AVS. By accurately visualizing the RAV and determining its location, CT imaging can facilitate successful cannulation and improve the efficiency of the AVS procedure. This ultimately contributes to better patient outcomes in the management of primary aldosteronism, reinforcing the recommendations made by expert consensus statements [7].

## 5. Conclusions

Pre-procedural inspiratory CT effectively visualizes the RAV in 99.2% of patients and provides essential anatomical guidance for AVS planning. However, operators must systematically account for the respiratory motion-induced height discrepancy to optimize procedural success. By proactively accounting for this discrepancy, operators can minimize the procedure time, reduce the contrast usage, and enhance the overall success rate of adrenal vein sampling.

## Figures and Tables

**Table 1:** Patient and procedure characteristics.

Characteristic	Value
Number of patients	133
Age (y), median (range)	53 (20–73)
Sex	
Male, *n* (%)	79 (59.4)
Female, *n* (%)	54 (40.6)
RAV selectivity index, median (range)	29.3 (5.1–96.6)
Catheter type used	
C1, *n* (%)	80/133 (60.2)
SIM1, *n* (%)	35/133 (26.3)
MIK, *n* (%)	13/133 (9.8)
SIM2, *n* (%)	5/133 (3.7)

## Data Availability

The data presented in this study are available on request from the corresponding author. The data are not publicly available due to privacy reasons.

## Abbreviations

The following abbreviations are used in this manuscript:

PA	Primary Aldosteronism
AVS	Adrenal Vein Sampling
RAV	Right Adrenal Vein
IVC	Inferior Vena Cava
PACS	Picture Archiving and Communication System
SR	Selectivity Ratio
